# EDUCATION AND COGNITIVE ABILITY: EXPLORING DIRECT AND INDIRECT EFFECTS IN THE UNITED STATES AND MEXICO

**DOI:** 10.1093/geroni/igac059.2954

**Published:** 2022-12-20

**Authors:** Joseph Saenz, Amina Khan

**Affiliations:** University of Southern California, Los Angeles, California, United States; University of Southern California, Los Angeles, California, United States

## Abstract

Education positively relates with cognition, which may be explained by enhanced cognitive reserve. However, education may also impact cognition indirectly by improving health, health behaviors, and life-course socioeconomic status (SES). This analysis explores the associations between education and cognition in the US and Mexico and quantifies the extent to which associations are direct versus indirect through health and SES. We use data from two studies: the MexCog in Mexico (n=2,042) and US Harmonized Cognitive Assessment Protocol (HCAP, n=3,267). Cognitive domains included Memory, Executive Function, Language, Visuospatial, and Orientation. Karlson-Holm-Breen (KHB) methods were used in linear regression models to quantify how much of the associations between years of education and cognitive domains were direct versus indirect through chronic conditions, income, wealth, smoking, and exercise. In regression models, years of education related positively with all cognitive domains in both studies, even when controlling for health and SES. KHB mediation analyses suggested that most of the education-cognition association was direct. In MexCog, estimates of the percent of the education-cognition association that was indirect through health and SES ranged from 4.17% (Memory) to 5.15% (Executive Function). In HCAP, indirect effects ranged from 8.95% (Orientation) to 12.15% (Language). Education was associated with better cognitive abilities in the US and Mexico regardless of cognitive domain or adjustment for late-life health and SES. Results suggested that education primarily related with cognition directly and that effects of education on cognitive abilities are not eliminated by reducing educational disparities in the late-life health and SES factors we analyzed.

